# Influence of Strength Programs on the Injury Rate and Team Performance of a Professional Basketball Team: A Six-Season Follow-Up Study

**DOI:** 10.3389/fpsyg.2021.796098

**Published:** 2022-02-01

**Authors:** Toni Caparrós, Javier Peña, Ernest Baiget, Xantal Borràs-Boix, Julio Calleja-Gonzalez, Gil Rodas

**Affiliations:** ^1^National Institute of Physical Education of Catalonia, University of Barcelona, Barcelona, Spain; ^2^Sport Performance Analysis Research Group (SPARG), University of Vic – Central University of Catalonia (UVic-UCC), Vic, Spain; ^3^Sport and Physical Activity Studies Centre (CEEAF), University of Vic – Central University of Catalonia (UVic-UCC), Vic, Spain; ^4^Universitat de Vic – Universitat Central de Catalunya, Barcelona, Spain; ^5^Physical Education and Sports Department, Faculty of Education and Sport, University of the Basque Country (UPV/EHU), Vitoria-Gasteiz, Spain; ^6^FC Barcelona Medical Services, Sant Joan Despí, Spain

**Keywords:** individualization, periodization, load monitoring, force, time to peak velocity, muscle injuries

## Abstract

This study aims to determine possible associations between strength parameters, injury rates, and performance outcomes over six seasons in professional basketball settings. Thirty-six male professional basketball players [mean ± standard deviation (SD): age, 30.5 ± 4.7 years; height, 199.5 ± 9.5 cm; body mass, 97.9 ± 12.9 kg; BMI 24.6 ± 2.5 kg/m^2^] participated in this retrospective observational study, conducted from the 2008–09 to the 2013–14 season. According to their epidemiological records, each player followed an individual plan designed within different strength training programs: Functional (*n* = 16), Eccentric (*n* = 8), or Resistance (*n* = 12). Seven hundred and fourteen valid records were obtained from 170 individual strength tests during 31 sessions. Tests performed were leg press, squat, and jerk. Parameters recorded were force, power, velocity, peak velocity, and time to peak velocity for strength; time loss injury and muscle injury for injury rate; and games won, games lost, and championships for performance outcomes. All the strength variables and injuries are independent of the strength programs (*p* < 0.01). The correlation analysis showed very significant relationships between muscular injuries and time to peak velocity (*r* = 0.94; *p* < 0.01), significant relationships between force and games lost (*r* = 0.85; *p* < 0.05), and muscular injuries with games lost (*r* = –0.81; *p* < 0.05) per season. Mean values per season described a possible association of force, time to peak velocity, and muscular injuries with performance outcomes (*R*^2^ = 0.96; *p* < 0.05). In this specific context, strength variables and injury rate data show no association with a single type of strength training program in this cohort of high-performance basketball players.

## Introduction

Basketball is a team sport with a complex nature (Mallo, 2020), and the competitive outcome is based on multiple performance factors. Therefore, it is almost impossible to know to what extent the physical, technical, tactical, or emotional aspects have contributed to athletic performance. Thus, numerous studies categorize relevant performance factors in the National Basketball Association (NBA) (Huyghe et al., 2021), the EuroLeague (Paulauskas et al., 2018), prestigious European championships, such as the Spanish “Liga ACB” (García et al., 2014), or even National Team competitions like the FIBA World Cup (Zhang et al., 2020), or the Olympic Games (Sampaio et al., 2010). However, these studies focused exclusively on analyzing the technical-tactical aspects, acknowledging that physical, physiological, or mental factors contribute to the player performance but without studying their specific impact on the game.

Other studies have adopted a different approach, monitoring, and assessing the impact of different training methods on basketball players’ physical fitness, the impact of physical fitness on the execution of technical and tactical abilities, or the significance of injuries throughout a sports season at an individual and team level. For instance, Naclerio et al. (2013b) observed that high volume resistance training (three sets per exercise and nine sets per muscle group) was the best approach to increase strength in college team sport athletes with no previous resistance training experience during pre-season, while low volume (one set per exercise and three sets per muscle group) seemed to be an interesting in-season strategy for maintaining strength and enhancing lower-body average power. The effects of these different programs were assessed *via* one repetition maximum (1 RM) and maximal average power (AP) on the bench press, upright row, and squat exercises using progressive tests. According to a study comparing professional and semiprofessional male basketball players, a standard preparation period (5–7 weeks, with athletes practicing 5–12 times a week, with 60–120 min practices) can induce improvements in professional players in abilities such as change of direction (COD). However, minimal differences between professional and semi-professional players were reported in the countermovement jump (CMJ) (Ferioli et al., 2018). On the other hand, a meta-analysis published in 2016 on strength training in healthy basketball players highlighted that interventions using external loads and even bodyweight exercises positively affected vertical jump ability (ES: 0.78 LARGE with 95% CI: 0.41, 1.15) (Sperlich et al., 2016). The effect of different circuit-training protocols in vertical jump height and peak power, horizontal jump distance, 3-points percentage, bench-press power output, RSA total and ideal time, and agility *T*-Test in semiprofessional basketball players has also been analyzed (Freitas et al., 2016). The authors found no changes in performance in the group participating in power circuit training (45% 1 RM), while the group using a high-resistance circuit training format (6 RM) presented decrements after 3 weeks. Plyometric training also seems a suitable training method to enhance muscle power, linear sprint speed, change-of-direction speed, balance, and muscle strength in basketball players, according to a recently published meta-analysis (Ramirez-Campillo et al., 2020). Similarly, Santos and Janeira (2012) demonstrated that a 10-week in-season resistance training program with moderate volume and intensity loads significantly increased vertical jump (*p* < 0.05) and medicine ball throw (*p* < 0.05) performances in the experimental group as compared to controls. Nevertheless, the study was conducted on twenty-five young male basketball players.

All the physical capacities improved by the strength and conditioning (S&C) practices mentioned so far are relevant, as research shows a translation between fitness and technical performance. Analyzing this relationship is relevant in highly complex sports such as basketball if we want to understand the reasons why the improvement of physical abilities can to some extent be associated with optimal competitive performance. Thirty-eight first division players from Bosnia-Herzegovina showed an association *via* multiple regression between higher fatigue resistance and free throw performance, preplanned agility, countermovement jump, and fatigue resistance with the two-point shot D2 (*R* = 0.44; *p* = 0.03), and countermovement jump, medicine ball toss, and anaerobic endurance with the three-point shot accuracy (*R* = 0.39; *p* = 0.03) (Pojskic et al., 2018). However, we must clarify that the study evaluated technical performances outside of an actual competitive situation using static and dynamic shooting tests. According to a study with twenty-eight first division basketball players from Turkey, the type of training also seems a significant issue. The results showed that one of the study groups, under a functional training program (core strengthening and specific basketball task-related exercises with/without equipment) lasting 20 weeks with a frequency of two sessions per week, significantly improved upper and lower body strength, flexibility, vertical jump ability, and T-drill agility scores when compared to a control group following a more traditional strength training program consisting of free-weight and machine-based exercises (Usgu et al., 2020). The use of ecological tests to measure the benefits of the different training programs is also relevant since some articles highlight the absence of association between strength measures and results from field tests (Alemdaroglu, 2012). Regarding the use of different exercises, an interesting piece of research surveying soccer, basketball, handball, volleyball, indoor soccer, and field hockey elite Spanish teams (Reverter-Masía et al., 2009) observed that only handball and volleyball coaches used Olympic lifts consistently. Many single-joint exercises were used by indoor and outdoor soccer teams and, especially, basketball teams. Basketball and handball were the sports mainly using weighted squat jumps. Similarly, a study surveying 20 NBA S&C coaches (69% response rate) found that all the respondents used strategies to develop the range of motion, followed some periodization, with Olympic lifts being used by 95% of the coaches (*n* = 19), and reporting that the squat or its variations were used by many of the teams, and all of them employing plyometrics in their practices (Simenz et al., 2005). Therefore, research demonstrates that the variability of methods used in the physical training of team sports is substantial and relevant differences between scientific evidence and what professionals do in the “real world” also exist.

Injuries in basketball are inevitable, as in any other discipline. Starkey reported that ankle sprains were the most frequent injury (9.4%), followed by patellofemoral inflammation (8.1%), lumbar strains (5.0%), and knee sprains (2.3%) in NBA players. Drakos et al. (2010), in a similar fashion, developed a 17-year longitudinal study in the same league, finding again that ankle sprains were the more recurrent medical issue (13.7% of the cases observed), while patellofemoral inflammation was the leading cause of more missed games (17.5%). According to the authors, professional NBA players undergo a high rate of game-related injuries. Contrarily, Rodas et al. (2019), in a study considering injury in an elite Spanish basketball club competing at the highest level of national and European leagues over nine seasons, found that muscle injuries (21.2%) were more commonly observed compared with ankle sprains (11.9%). Thus, prevention and therapeutic approaches to injuries in professional basketball settings are somewhat relevant.

Different authors have also analyzed the relationship between injuries, strength levels, workloads, and team-sport performance. Caparrós et al., 2014 found relations between squat strength (force), better performance (scored points) (rho = –0.81; *p* < 0.05), and fewer time-loss injuries (TLI) (rho = 0.82; *p* < 0.05) in a prospective study conducted on 12 Spanish professional male basketball players. Another study led by the same first author, but this time on NBA players, found that athletes under lower external loads were more prone to TLI (Caparrós et al., 2018). As some leagues present demanding competition schedules (McLean et al., 2018), the benefits of applied load management processes and different monitoring strategies seem relevant to protect professional players’ physical integrity (Burgess, 2017). It is hypothesized that strength programs may reduce inter-limb asymmetries, a well-known internal risk factor for injuries (Bahr and Holme, 2003), limiting physical performance (Šarabon et al., 2020) and availability (Gabbett et al., 2018b).

Despite available research, to the best of our knowledge, there has been a limited attempt of examining the relationships between the use of different individualized strength training programs, injury rates, and competitive achievements in basketball longitudinally. This study aimed to determine possible associations between strength parameters, injury rates, and performance outcomes over six seasons in a European professional basketball team.

## Materials and Methods

### Study Design

A retrospective observational study was carried out during six seasons in a professional basketball team (FC Barcelona) that played four main competitions every season. The data collection took place from 2008–09 to 2013–14, and players were allocated in three different strength training groups (functional, eccentric, or resistance training program) on each season, depending on their medical record. The measurements included individual strength assessments for each training group at the beginning and end of the mesocycles and team performance outcomes assessments per season. Baseline medical information was recorded from all participants at the beginning of each season through the FC Barcelona periodic health examination protocol. The protocol consisted of basic medical information (history), anthropometric data (age, height, weight, and ethnicity), physical examination, spirometry, basal 12-lead electrocardiography (ECG), submaximal cardiovascular exercise testing (with ECG and blood pressure monitoring), and cardiac echocardiography. Once a season started, various parameters potentially related to the type and frequency of musculoskeletal injuries (e.g., mechanism of injury) were collected. Athlete exposure and other variables, such as playing position, were recorded. We also collected clinical information and data related to the type of injury, TLI, medical attention (MA), and return to play (RTP) (Hägglund et al., 2005). Before implementing the strength training programs, all participants were required to perform familiarization tests and sessions. The S&C coach recorded all strength and performance data (TC). The Team Physician (GR) was responsible for diagnosis and RTP decision-making for every injury and recorded all the injuries included in the current investigation. Data were recorded daily after every practice and game. All participants took part in another retrospective study previously published (Caparrós et al., 2016).

### Participants

Thirty-six professional basketball players [mean ± standard deviation (SD)]: age, 30.5 ± 4.7 years; height, 199.6 ± 9.5 cm; body mass, 97.9 ± 12.9 kg; BMI 24.6 ± 2.5 kg/m^2^) from a Spanish basketball club (FC Barcelona) participated in this study. Thirty-two of them were Caucasian, and four were African American. Regarding their playing roles, 10 were guards, 11 were forwards, and 15 were centers. Two players played at the team during the six seasons of this follow up; 1 during 5; 5 players over 4 seasons; 4 over 3, 11 over 2, and 36 of them took part in at least 1 season (1.7 ± 1.2 seasons per player) with a mean value of 13.0 ± 0.9 players per season. The inclusion criteria for all subjects required each participant to be part of the FC Barcelona professional team roster during a complete season and aged >18 years, not being involved in a TLI rehabilitation process, and not to change between training methods during the same season. During the study, 8 of the 44 starting participants were not able to meet the inclusion criteria. All the players and the club (FC Barcelona) were informed of the risks and benefits of the study and gave written informed consent to participate in this study. Players were allowed to decline the inclusion of their data. The study was conducted following the ethical principles for biomedical research with human beings, established in the Declaration of Helsinki of the World Medical Association (amended in 2013), and it was approved by the club Board of directors and the Research Ethics Committee of the University of Vic-Central University of Catalonia (favorable report available upon request).

### Strength Measurements

Strength assessments were carried out at the beginning of the first mesocycle and at the end of each mesocycle to evaluate each period’s initial and final state (Bangsbo et al., 2006). During the analyzed period, tests were conducted during the training sessions and were non-invasive (Bangsbo et al., 2006) using the main exercises of each program. They were performed during introductory microcycles (Naclerio et al., 2013b), and depending on the strength program and seasonal periodization, the test exercises were: single leg press (LP) (Cuadrado Sáenz et al., 2009) for the eccentric (ECC) and resistance (RES) programs and double-leg squat (SQ) (Caparrós et al., 2014) and jerk (JK) (Andújar Gutiérrez et al., 2015) for the functional (FUNC). Tests were carried out during the morning sessions after a full rest day, beginning with a warm-up consisting of 8 min of submaximal general physical activity, lumbopelvic analytic protocol, and joint mobility. Parameters recorded were force (F), power (P), velocity (V), peak velocity (pV), and time to peak velocity (tpV). They were evaluated indirectly through data gathered using a linear encoder with an accuracy < 0.075 mm (MuscleLab PFMA V.4000e, Ergotest Innovation AS, Norway) (Porta-Benache et al., 2010). Every player performed four series of each exercise with a progressive weight increase of 10 kg and a decreasing number of repetitions in each series (12, 10, 8, and 6) (Naclerio et al., 2013a). Weights were individualized (Bangsbo et al., 2006) and could vary in each test (depending on the previous results and the periodized program), but there was always a minimum of two loads equal to the previous test, and the reference weight was the same throughout the whole season. The variables analyzed (F, P, V, pV, and tpV) in each exercise (LP, SQ, and JK) were determined to be reliable showing the following Guttman’s Lamba 6 (G6) and coefficient of variation (CV) interval values at different weights: 115 kg [40–50% of 1 repetition maximum (1 RM)]; for single LP (G6 95 % confidence interval [CI] = 0.86–0.99; CV 95% CI = 0.01–0.29), 90 kg (40–50% 1 RM) for SQ (G6 95% CI = 0.94–0.99; CV 95% CI = 0.01–0.24), and 35 kg (40–50% 1 RM) for JK (G6 95% CI = 0.81–0.94; CV 95% CI = 0.05–0.26).

All the players underwent assessments as part of their training routine, and therefore the possibility of a player being injured because of the participation in the study was not considered. The S&C coach carried out the assessments, and all the players were familiar with the technique and protocol. To ensure that the testing was carried out consistently, the acoustic feedback provided by the encoder software was enabled during the execution of the tests. It was used to determine the minimum individual power level to be achieved.

### Injury Measurements

To monitor injuries, we followed the model proposed by Hägglund et al. (2005), as well as the premises of Fuller et al. (2006). The study focused on TLI that caused absence from practices, workouts, or games, and among these, the group of muscular injuries (MI)—rupture, tear, strain, cramps or tendon ruptures, tendinosis or bursitis—were followed up.

### Performance Outcomes Measurements

Five Team performance outcomes were considered: the Spanish Super Cup, the King’s Cup, the Euroleague Final Four qualification stage, the Euroleague Final Four, and the Spanish League (ACB). Games won (GW), games lost (GL), and championships won (CW) were recorded on each season (Caparrós et al., 2016).

### Periodization

**FIGURE 1:**
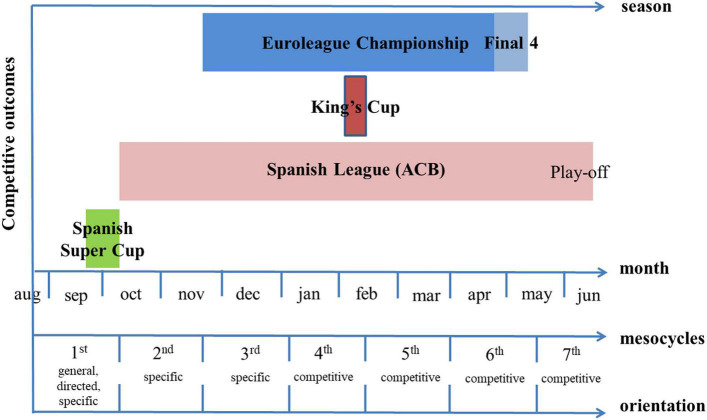
Timeline of season competitive outcomes, mesocycles, and mesocycle orientation.

To achieve the competitive goals, every season was divided into seven mesocycles, always considering the competition calendar. The first performance outcome fell within mesocycle 1 (Spanish Super Cup). Mesocycle 2 focused on the start of the regular season of the Spanish League (ACB) and the Euroleague season. Most of the first phase of the regular competitions takes place during Mesocycle 3. Mesocycle 4 included the second performance outcome (King’s Cup). Mesocycle 5 covered the second phase of the regular competitions and the Euroleague playoffs (third outcome). Mesocycle 6 ended with the Euroleague Final Four (fourth outcome), and Mesocycle 7 comprised ACB playoffs for the championship as the fifth performance outcome (see Figure 1).

The duration of each season was 43 ± 2 weeks. Workload planning, both conditional and technical/tactical, was designed with as similar a structure as possible between mesocycles, with a duration of 6 ± 1 weeks. Mesocycle periodization followed the blocked periodization model (Issurin, 2008), and the progression of contents was divided into four orientations (general, directed, specific, and competitive) (Schelling and Torres-Ronda, 2013).

**FIGURE 2:**
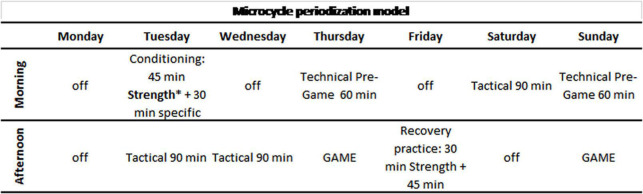
Microcycle periodization model; ^∗^ test sessions.

The design of the mesocycles was adapted to the game days, two per week during the regular season phases (31 weeks), except 5 ± 2 weeks with a single game. During the ACB and Euroleague playoffs, and in the King’s Cup (8 weeks), three games were played weekly. The usual structure of each of the microcycles in each mesocycle was as follows: 1 day or morning session free with a recovery session in the afternoon; Monday and Tuesday: double session with strength and individual strength workouts in the morning and tactical session in the afternoon; Wednesday: tactical session in the afternoon; Thursday: pre-game session in the morning and game in the afternoon; Friday afternoon: recovery and joint technical/tactical session; Saturday morning: tactical session; and Sunday: game (with a pre-game session if it was in the afternoon) (see Figure 2). In addition, each player had an individualized preventive program, a strength workout, or practice to be performed before joint practices or during free days. Players with a lower competitive load had a compensatory practice on Monday morning (Gabbett, 2016; Caparrós et al., 2018).

### Strength Programs

**FIGURE 3:**
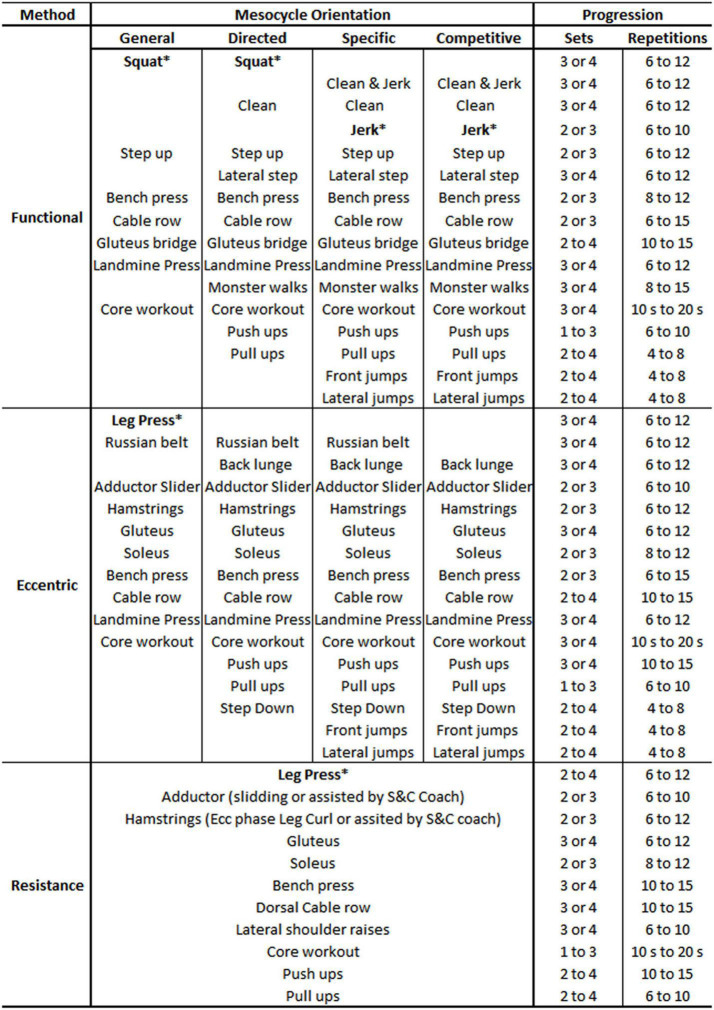
Main strength exercises and progression by method and mesocycle orientation; ^∗^test exercises.

An integrative strength training program was designed for each player (Schelling and Torres-Ronda, 2016). In the light of their medical history, the players were distributed into three distinct workgroups. Those with the fewest constraints participated in the FUNC program (Schelling and Torres-Ronda, 2016), consisting of a multi-joint exercise routine, easily transferable to competitive play (Pojskic et al., 2018). These routines were performed with free weights, bodyweight exercises, medicine, weight balls, resistance bands, and mini bands. Those players whose injury records showed a history of tendon or muscle injuries were placed in the ECC program, which included both multi-joint and analytic exercises and was eminently preventive, emphasizing the eccentric component of the exercises (Roig Pull and Ranson, 2007; Peña et al., 2017). The tools used for this program were strength machines, own bodyweight, medicine ball and weight balls, and resistance bands and mini bands. Finally, those who had chronic joint injuries or were older were assigned to the Resistance (RES) program, oriented to specific muscle groups (Naclerio et al., 2013b). Tools used for this program were weight machines, own body weight, and resistance bands and mini bands. Subsequently, depending on their age and playing characteristics, the exercise program, progression, and workload were adapted individually. The program’s degree of specificity (Wen et al., 2018) and the volume and intensity of work were determined by the time of the season and by the orientation of the previous mesocycles. Therefore, strength work was integrated with the team’s technical and tactical needs according to the competition calendar (McLean et al., 2018; see Figure 3).

### Statistical Methods

Data are presented as mean ± standard deviation (SD). After performing a central tendency descriptive study and considering the non-normality of the sample, the Kruskal–Wallis test was used to evaluate the effects of the strength training program (independent variables) on dependent variables (strength and injury rate parameters). For this purpose, we used the values (F, P, V, pV, and tpV) of the best repetition in each series of each test (LP, SQ, and JK) recorded. A Dunn-Bonferroni *post hoc* test was performed in turn. Intrasession reliability of measures was determined using the Guttman’s Lambda 6 test with 95% confidence intervals (Oosterwijk et al., 2016). The results were weighted to take account of the number of players in each program. Subsequently, and considering the normality of the average values by season, it was performed Pearson’s rho correlation analysis between the variables of the best repetition in each test for the strength, injury, and performance outcome variables. The correlation magnitude was defined according to Hopkins’s criteria (Hopkins, 2002): random: 0–0.09; low: 0.10–0.29; moderate: 0.30–0.49; large: 0.50–0.69; very large: 0.70–0.89; nearly perfect 0.90–0.99; perfect: 1. Finally, the multiple linear regression analysis used performance outcomes as the dependent variable, whereas strength and injury rate parameters operated as independent predictors. The statistical analyses were performed with JASP software version 0.11.1 (The Jasp Team, Amsterdam, Holland). The level of significance was set at *p* < 0.05.

## Results

The 36 players were distributed between the strength training programs: RES: 12; ECC: 8; FUNC: 16. Seven hundred fourteen test valid records were obtained (27.5 ± 3.5 per player), of which 111 were from the RES group, 132 from ECC, and 471 from FUNC. The records were made during 170 individual tests in 31 sessions (5.2 ± 1.9) days of tests per season and (4.9 ± 2.2) tests performed per player. Of these tests, 26 were LP for the RES group, 12 were LP for ECC, 108 were SQ for FUNC, and 24 were JK for FUNC. There was a relevant difference between the numbers of test records in different seasons. The average was 119 records per year, going from a maximum of 228 in the second season (2009–10) to a gradual decline, reaching 29 in the last season (2013–14).

### Strength Programs

**FIGURE 4:**
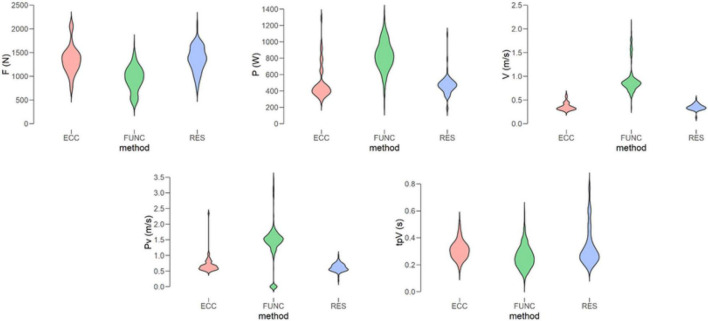
Absolute values of Force (F), Power (P), Velocity (V), peak Velocity (pV), and time to peak Velocity (tpV) by strength training method (Eccentric—ECC—Functional—FUNC—or Resistance—RES). N, newtons; W, watts; m/s, meters per second; s, seconds.

Regarding the strength values recorded for each program, RES showed the highest F (1196.1 ± 356.8 N) compared to the FUNC with the lowest values (964.3 ± 266.2 N). FUNC showed the highest values for P (842.5 ± 183.1 W), V (0.92 ± 0.29 m/s), and pV (1.39 ± 0.58 m/s), and a lower tpV (0.25 ± 0.08 s). RES showed the worst values in all these cases, except in tpV, where it was ECC (see Figure 4).

Statistically, the Kruskal-Wallis test enabled us to determine the independence (*H* = 43.69–146.61; *df* = 2; *p* < 0.001) of all the strength variables (F, P, V, pV, and tpV) according to the test performed and considering all the strength programs. The *post hoc* test, in turn, also determined that these differences were present between each of the variables in every training group (z: –10.37 to 7.41; wi: 197.77–467.29; wj: 256.26–467.09; *p*_*Bonf*_ < 0.05).

### Injuries

A total of 149 TLI were recorded during the six seasons, with averages of 24.7 ± 7.6 TLI and 6.0 ± 1.9 MI per season. An incidence of 37 TLI in the 2012–13 season stands out as the highest value, with a maximum of 9 MI, in contrast to the 2011–12 season with 17 TLI, only 3 were MI. In terms of mesocycles, the third produced the highest number of injuries (58), followed by the second (24), fourth (14), fifth (13), seventh (11), first (10), and sixth (6).

**FIGURE 5:**
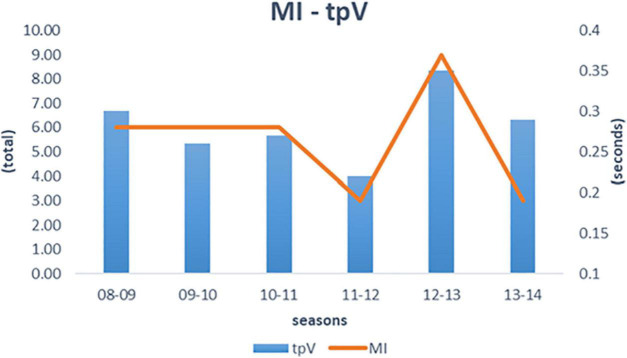
Relationship of Muscle Injuries (MI) and mean season time to peak Velocity (tpV), by season. 08–09: 2008–2009; 09–10: 2009–2010: 10–11: 2010–2011: 11–12: 2011–2012; 12–13: 2012–2013; 13–14: 2013–2014. s, second.

No significant differences were observed in the number of injuries according to the training group, so they should not be determined by this factor. Differences did emerge between seasons (*H* = 36.21; *df* = 5; *p* < 0.001) and mesocycles (*H* = 65.01; *df* = 6; *p* < 0.001). The *post hoc* test highlighted differences between the 2012–13 with the rest of the seasons (z: –4.57 to 4.13; w_i_: 315.62–385.65; w_j_: 315.62–432.3; *p*_*Bonf*_ < 0.01) (see Figure 5). Regarding the mesocycles, the same test showed differences between the third and the other six (z: –6.4 to 6.05; w_i_: 322.86–439.54; w_j_: 322.58–434.54; *p*_*Bonf*_ < 0.001).

### Team Performance Outcomes

A total of 425 games were played, of which 342 were won (80.5%) and 83 lost (19.5%), with an average of 57.0 ± 4.3 games won (GW) per season, reaching a peak of 63 in the 2013–14 season, and 13.8 ± 4.6 games lost (GL) per season, the highest value was 21 in 2012–13. In all, 16 competitive objectives were attained, an average of 2.7 ± 0.9 per season, outstanding among which were the four performance outcomes in 2009–10 (Super Cup, King’s Cup, Final Four qualification, and Euroleague Championship). Three were achieved during the 2010–11 and 2011–12 seasons, and two in the other seasons.

### Relationships Among Variables

The correlation analysis using Pearson’s rho test showed very significant (nearly perfect) relationships between MI and tpV (r: 0.94; *p* < 0.01) (see Figure 5) and significant (very large) relationships between F and GL per season (r: 0.85; *p* < 0.05) and also between MI and GL per season (r: –0.81; *p* < 0.05).

A multiple linear regression analysis was used to select the most promising independent variables (strength and injury rate parameters) to determine performance outcomes. The procedure revealed that F, tpV, and MI parameters together accounted for 92% of the variation of performance outcomes over the six seasons (*r* = 0.98, *r*^2^ = 0.97, adjusted *r*^2^ = 0.92, *p* < 0.01).

## Discussion

The present longitudinal study aimed to analyze potential associations among three strength training programs, team performance, and injury rates during six seasons in a top basketball professional team that included thirty-six male players. Among others, the most critical findings in this specific context are that strength variables and injury rate are independent of the training program. Finally, strength variables such as F, tpV, and MI could be associated with team performance outcomes. However, the team periodization might be designed and interpreted according to the individual players’ needs, competitive schedule, and team goals of each different season.

The competition calendar determines team periodization (Ireland et al., 2019), with a very high traveling frequency required to compete in elite professional sport (Calleja-Gonzalez et al., 2020). In this scenario, the design of both strength programs and their workload should be adapted to each player’s individual needs and characteristics (Rogalski et al., 2013). Concretely, during the six seasons analyzed, the players studied were distributed in three types of strength programs precisely based on their individual profiles (Gabbett et al., 2014). No differences emerged among the recorded variables, either of strength or susceptibility to injury, allowing to assess this distribution positively and design tailored to each player (Bogdanis et al., 2007). For this purpose, it is necessary to monitor, as far as possible, and in a specific and non-invasive manner (Bangsbo et al., 2006), all the available performance, physical fitness, and health parameters from a holistic approach (Nagorsky and Wiemeyer, 2021). Using a comprehensive framework for sports training with a performance model integrating insights from game research and sport science (Calleja-González et al., 2018) the aim is to be fully available for competitive play (Gabbett et al., 2018b). Monitoring of load management allows to adapt individual programs (Fernández et al., 2021), to competition demands, but involving players and staff in common team objectives (Thorpe et al., 2017) and combine competition demands and each member of staff (Gabbett et al., 2018a).

Overall, all the strength variables analyzed behaved independently of each other. Periodization was different every season, given that although the coach and staff were the same, the entire roster was not. In turn, the programming and load design were adapted to the determinants of a highly competitive context: changing squad, with some older players; more games for a new Euroleague schedule from the 2011–2012 season; and an increasing pressure to maintain the team performance outcomes success. The data can be well-contextualized in the team’s current competitive situation, allowing each season and programming unit to be interpreted independently. Periodization will depend on multiple factors (Aoki et al., 2017), and each season must be analyzed and all the contents tailored to specific needs (Stone and Steingard, 1993).

From the 2011–12 season onward, the number of games in European competition increased, and this meant going from having between 12 and 14 weeks with a single game to at most 4. At that point, conditioning had to be oriented more to recovery than to accumulation (Fox et al., 2018) managing strength work now focused on intensity rather than volume (Naclerio et al., 2013b). Up till then, the first day of the week in the gym was performed with general or directed contents, and second for more specific ones. But from then on, the second weekly session was predominantly for recovery (Calleja-González et al., 2016), so the first had to include more specific contents during the period of competition (Fox et al., 2020), with the aim to perform higher P, V, and pV values (Santos and Janeira, 2012). The fact is that the new calendar entailed a reduction in the accumulation period, additionally to the constraint of competing for the first competitive goal at the end of the preseason (Doeven et al., 2020). In this sense, the F levels could not be adopted as before, representing a physical limitation (Šarabon et al., 2020). Greater specificity may conditionally be assumed (Fernandes-da-Silva et al., 2016), but it can be related to other issues such as performance (Gabbett et al., 2018a) and susceptibility to injury (Hulin et al., 2016). Each phase of the season presents different demands (Ferioli et al., 2018): the first phase of three mesocycles was oriented toward F work, with the subsequent management of P and qualitative and specific issues at the end of the season (Ferioli et al., 2021). This could be related to susceptibility to injury and observed during the third mesocycle (accumulating as many as 37 injuries), a period that coincides with an overload of competition and training, as well as periods of acute overload (spikes) (Hulin et al., 2014) at a conditional level. Correct data interpretation would lead to moderate this workload in this mesocycle to aid recovery (Terrados et al., 2019). For this purpose, the preceding mesocycles are essential in the appropriate management of chronic workload (Gabbett, 2016), and monitoring weekly changes during the in-season phase should help to adjust acute workload that may predispose players to unwanted spikes (Paulauskas et al., 2019).

In a previous study with this sample (Caparrós et al., 2016), TLI was not related to the competitive outcome. However, in this case, the correlation analysis, now including conditional factors, does identify clear relationships between MI and F and GL in the season (*p* < 0.05)—which in turn are related to the achievement of championships as team performance outcomes (*p* < 0.05). The fact is that during the first three seasons, the development of F in the first mesocycles made it possible to take on conditional levels that enabled players to reach a competitive fitness state (Speranza et al., 2015) and with the squad members available in all competitions. Nevertheless, during the last three seasons (from 2011 to 2012), the exercises’ orientation was more specific on a more congested calendar. Subsequently, these are associated with greater susceptibility to injury in this specific context, especially muscle injury, which would be closely related to lower F values (Malone et al., 2019) or worse recovery, reflected in variables of a qualitative nature that deteriorate, such as pV (Serpiello et al., 2011). These factors are associated with shorter preseasons (Killen et al., 2010) and a fuller competitive calendar, noting two different trends: first, there is the highest injury incidence during the third mesocycle. Players could have reached this point in a competitive state of fitness but with less capacity for recovery from these exertions at this point of the season (Killen et al., 2010). And second, lower injury rates are on seasons with best tpV values. Load management must balance recovery strategies and optimal strength training, avoiding low chronic workloads (Malone et al., 2019) too early in the season.

It is essential to note the association among F, tpV, and MI (*R*^2^ = 0.96), but it needs to be put into context. The proposed model establishes that in this specific context, the three variables determine performance outcomes. The optimal chronic strength values (Caparrós et al., 2014) and the specific details of the proposal need to be determined for each player (Ferioli et al., 2021). The tpV value and its possible relationship to muscle recovery (Serpiello et al., 2011) and better states of fitness (Fernandes-da-Silva et al., 2016) must be adequately monitored throughout the in-season (Gabbett, 2016; Paulauskas et al., 2019). These two factors, in turn, could determine muscle injury. Injuries may continue not to impede competing in championships. However, correct management and monitoring of variables such as F and tpV, and consequently a reduction in muscle injuries (Rodas et al., 2019), will make it possible to have a roster in its full potential to achieve the maximum number of competitive goals, as happened in the seasons from 2009–10 to 2011–12.

The present study presents limitations inherent in a sporting and competitive context, despite the wealth of data related to the study period, players in the sample, and team performance outcomes. First, and from a global vision, the possibility of comparing this longitudinal study with others would allow us to determine the applicability of the results. Second, and for this specific case, having different tests does not allow us to obtain consistent results, but that was not the intention in this research. Each workgroup and player must be assessed independently to adopt the same objectives simultaneously as a team. In this connection, given these specific sporting processes, not all the players were monitored using all the tests; in some cases, they were injured. Third, and related to the number of tests, a gradual decrease occurred as the seasons passed. Nevertheless, primarily, the reduction in the quality of monitoring must be assessed and, once again, interpreted: the increase in the number of games reduced the number of sessions that included exercises for recording data. And especially in the last two seasons, although competitive objectives were undertaken, the number of games lost was higher, and consequently, competitive pressure did not make it easy for the staff to manage data collection. Neither the seasons’ routine nor adverse competitive situations should give rise to a lack of rigor in any detail of the training process. These are precisely the situations in which greater importance should be given to monitoring (Burgess, 2017), to optimize the players’ performance as much as possible, monitoring the attainment of the objectives of periodization, and not overlooking risk factors (Gabbett et al., 2018a). However, the strength of this study is a six-season follow-up of top Euroleague players, to date not described, in the daily reality of the sports competition.

To conclude, in this specific context, strength variables and injury rate data show no association with a single type of strength training program in this cohort of high-performance basketball players. F, tpV, and MI showed association with team performance outcomes.

## Practical Applications

Performance (both team and individual) data and strength variables should be integrated in the workload monitoring process, with the aim to optimize training, individual availability for competition, and team performance outcomes. This process is longitudinal, and it is the staff’s responsibility to involve the players in it to understand their importance for improving their performance and health. Sports organizations and coaches should assess these data using this perspective. In turn, S&C and Sports Science professionals have more tools and experience available to manage this process in an ethical and minimally invasive manner, providing the rest of the staff and the players with concise and reliable information.

## Data Availability Statement

The raw data supporting the conclusions of this article will be made available by the authors, without undue reservation.

## Ethics Statement

The studies involving human participants were reviewed and approved by the Universitat de Vic-Universitat Central de Catalunya Research Ethics Committee. The participants provided their written informed consent to participate in this study.

## Author Contributions

TC and GR conceived and designed the research, analyzed and interpreted the data, drafted the article, and approved the final version submitted for publication. JP and EB analyzed and interpreted the data, and drafted the article. JP, EB, XB, and JC-G critically reviewed the article and approved the final version submitted for publication. All authors contributed to the article and approved the submitted version.

## Conflict of Interest

GR was employed by the FC Barcelona Medical Services. The remaining authors declare that the research was conducted in the absence of any commercial or financial relationships that could be construed as a potential conflict of interest.

## Publisher’s Note

All claims expressed in this article are solely those of the authors and do not necessarily represent those of their affiliated organizations, or those of the publisher, the editors and the reviewers. Any product that may be evaluated in this article, or claim that may be made by its manufacturer, is not guaranteed or endorsed by the publisher.
